# Food support provision in COVID-19 times: a mixed method study based in Greater Manchester

**DOI:** 10.1007/s10460-021-10212-2

**Published:** 2021-04-26

**Authors:** Filippo Oncini

**Affiliations:** Sustainable Consumption Institute and Department of Sociology, University of Manchester, Booth St W, Manchester, M15 6PB UK

**Keywords:** COVID-19, Food insecurity, Food aid, Food banks

## Abstract

COVID-19 has brought to light the severity of economic inequalities by testing the capacity of the poorest families to make ends meet. Food insecurity has in fact soared all over the UK, with many people forced to rely on food support providers to not go hungry. This paper uses a unique dataset on 55 food support organizations active in Greater Manchester during the first COVID-19 wave, and 41 semi-structured interviews with food aid spokespersons and stakeholders, to shed light on what they overcame, the complications and drawbacks of the food emergency response plan put in place. The results indicate that food aid organizations that remained open were surprisingly effective despite the growth in user demand and the decrease in volunteers. However, the necessity to maintain a timely supply food at all costs came with important drawbacks. The lockdown measures that followed COVID-19 not only affected the financial stability and management of the organizations, and the availability of food, but undermined the ways in which food support providers used to operate. Owing to physical distancing measures and to the increasing numbers of users, more or less intangible forms of support such as financial advice, empathic listening and human warmth were partially lost, probably when they were needed more than ever.

**Supplementary Information:**

The online version contains supplementary material available at 10.1007/s10460-021-10212-2.

## Introduction

The outbreak of COVID-19 and the lockdown measures that followed had a significant impact on our food systems and soon became a litmus test for the extreme inequalities of our times. Disruption to food supply chains, loss of income and livelihoods and uneven food price trends in localised contexts affected food security and nutrition all over the world (Clapp and Moseley 2020). On the production side, the crisis highlighted how much the EU’s agricultural output relies on exploitation of the migrant labour force, with governments quickly having to embark on policies to secure the food supply chain by granting temporary working permits to farm workers (Barling 2020; Neef 2020). Concurrently, while many families were stockpiling and increasing their expenditure on food (Dickinson 2020; Wilson 2020), the most vulnerable struggled to make ends meet. A recent survey by the European Food Banks Federation (FEBA 2020) showed that demand for emergency food assistance increased in every European country and even doubled in the UK. Although the UK Government (2020a) stressed ‘we are all in this together’ as a national motto to foster solidarity and cohesion, some were much further ‘in’ than the rest.^1^ Such universalistic claims not only fall short in recognising how diverse people’s capabilities to face a major crisis can be but also limit our possibility to rethink the right to good and sustainable food (Moragues-Faus 2020).

In fact, COVID-19 has brought to light the severity of economic inequalities by testing the capacity of poorer families to feed themselves. Food insecurity—the ‘limited, inadequate or insecure access to food due to financial resource constraints’ (Tarasuk 2001, p. 2)—has in fact soared all over the UK, putting to test the capacity of its retrenching welfare state. Recent estimates suggest that around 3 million people live in households where someone has to skip some meals, while food insecurity has quadrupled, extending to 16% of the British population (Loopstra 2020). This unprecedented crisis has prompted a massive response by the voluntary, community and social enterprise sector (VCSE), which has been at the forefront of food support provision since 2010 in light of the consequences of the austerity measures taken after the Great Recession (Loopstra et al. 2015; Garthwaite 2016a). The Trussell Trust, the Salvation Army and the Independent Food Aid Network (IFAN), to name just a few well-known food aid networks, all reported a growth in the number of requests for food parcels, and during the early days of the first lockdown called for food and monetary donations, fearing that these would probably drop (Barker and Russell 2020). For instance, IFAN food banks reported a 177% increase in the number of three-day emergency food parcels distributed in May 2020, comparing this with May 2019 (IFAN 2020).

Although the COVID-19 crisis tested the viability of many VCSE organizations, those that were able to remain open made gigantic efforts to keep pace with requests for food aid while observing physical distance requirements and maintaining logistics to reach all those in need. However, evidence on how food support providers have tackled the crisis is mostly anecdotal, or based on single case studies or time-sensitive accounts (e.g. Barker and Russell 2020; Power et al. 2020), and still lacking is a systematic analysis of their reaction to it. Seeking to fill this gap, this paper uses a unique dataset on 55 food support organizations based in Greater Manchester, and 41 semi-structured interviews with food aid spokespersons and stakeholders, to shed light on the obstacles they faced and the needs that emerged immediately after the epidemic’s peak. After a brief introduction on food support provision in the UK, I outline the strategy I used to collect data. I then present my findings, distinguishing between three different outcomes of the response to the crisis: successfully overcoming these obstacles, complications arising and restrictions. Overall, the results indicate that food aid organizations that remained open during the crisis were surprisingly effective despite the growth of user demand and the decrease in volunteers. However, the need to maintain the supply of food at all costs brought important drawbacks. The lockdown measures that followed COVID-19 not only affected the financial stability and the management of these organizations, and the availability of food, but actually undermined the ways in which most food support providers used to operate. In fact, owing to physical distancing and to the increasing numbers of users, more or less intangible forms of support such as financial advice, empathic listening and human warmth were partially or totally lost, probably when they were needed more than ever.

## Food support provision in the UK

The aftermath of the Great Recession has been characterised by the rise of food insecurity and food charities all over Europe (Lambie-Mumford and Silvasti 2020). In the UK, following cuts in welfare services, reforms of social security payments, benefit sanctioning, and growing disconnect between food prices and wages, food insecurity has reached staggering proportions (Hartfree 2014; Loopstra et al. 2016, 2018, 2019a; Reeves et al. 2017). In 2017, a study surveying a representative sample of the population in England, Wales and Northern Ireland found that 13% of participants lived in marginally food secure households and 8% lived in low or very low food secure households (Bates et al. 2017). The escalation in the number of people unable to access enough food to sustain an active and healthy life has been paralleled by the rise of food banks, charities that depend on monetary and food donations to provide emergency food parcels that can be taken home for free by people in need. For instance, the Trussell Trust Foodbank Network—the UK’s largest food bank organization—declared that between 2015 and 2020 the distribution of three-day emergency food parcels increased by 74%, from 1,112,395 to 1,900,122 (Trussell Trust 2020). Similarly, in a recent IFAN report Loopstra et al. (2019b) found that 75% of independent food banks started operating between 2010 and 2018, with over a third opening between 2012 and 2013.

Food banks have been widely criticized, as studies indicate that they are often unable to provide nutritionally adequate and balanced food, and that users often feel shame, stigma and embarrassment about being seen as incapable to provide for their families (Bazerghi et al. 2016; Garthwaite 2016b; Purdam et al. 2016). Moreover, despite being thought of as short-term safety nets, food banks are more and more used by those experiencing long-term deprivation as a complementary—and in some cases unique—form of social security (Bazerghi et al. 2016). At the same time, it is undeniable that without food banks many more people would experience hunger. And arguably, the depth of contemporary poverty in Britain would be less visible without them, as food banks also act as advocates for the development of a more equitable welfare regime and food system. Thus, the argument over the justification for food aid has stalled in an ethical cul-de-sac: their institutionalization partly legitimates a problematic transition from cash to food transfers, but at the same time could encourage and coordinate wider food justice campaigns (Williams et al. 2016). This ambivalence comes to light in the provisioning practices of many food banks: on the one hand, the use of intrusive assessment procedures, vouchers and referral systems contributes to the bureaucratisation of the sector, which is becoming more and more akin to a welfare apparatus, and to the perpetuation of the distinction between the deserving and the undeserving poor (Garthwaite 2016a, b; May et al. 2019); on the other hand, food banks also offer a ‘space of care’ characterised by acceptance, moral support, generosity, hospitality and advice, as well as a ‘liminal space’ of encounter’ between marginal groups, volunteers and staff members that could potentially lead to new political and ethical engagements (Cloke et al. 2017).

Possibly because of their spreading, media presence, data availability, and reports production, scholarly and public debates in Europe tend to concentrate on food banks as the prevailing form of food aid (Lambie-Mumford 2019; Lambie-Mumford and Loopstra 2020). Yet food aid is a heterogeneous field of assistance that encompasses ‘a range of large-scale and small local activities aiming to help people meet food needs, often on a short-term basis during crisis or immediate difficulty;’ (Lambie-Mumford and Loopstra 2020, p. 200). As well as food banks, we should include holiday hunger programmes, community kitchens, soup vans and food pantries (Lambie-Mumford 2019). In fact, the whole food-charity sector has expanded over the last decade. Although food insecurity and initiatives to ‘feed the poor’ have a long history in the UK (Glennerster et al. 2004), the scale, scope and professionalisation of food aid is a recent phenomenon, overlapping with the expansion of the third sector, which is increasingly presented as an institution that can fight social exclusion, reinvigorate civil society and encourage active citizenship (Fyfe 2005). Expansion, however, does not automatically ensure that organizations are able to offer the same services and food provision standards. Rather, different opportunities in accessing volunteers, donations and food redistribution schemes contribute to the creation of ‘an uneven geography of “scarcity” that reflects the unequal distribution of wealth at the local and regional level’ (May et al. 2020, p. 213).

The institutionalization of food aid has been further cemented by presenting (recovery of) food surplus and (reduction of) food poverty as a ‘win–win’ situation (Caplan 2017; Lohnes and Wilson 2018) capable of improving resource efficiency and environmental sustainability while addressing the needs of the most disadvantaged, thus responding simultaneously to two United Nations Sustainable Development Goals (SDGs): SDG 12 ‘Ensuring sustainable consumption and production patterns’, which aims to halve per capita food waste and reduce food losses; and SDG 2, ‘Ending hunger and ensuring access by all people, the poor and the vulnerable to safe, nutritious and sufficient food’ (Arcuri 2019; Galli et al. 2019).^2^

In this light, it is not surprising that the repercussions of the lockdown on the most disadvantaged have been almost entirely managed by charities and especially by food support providers. In a sense, there was no other apparatus as ready, skilled and qualified as the VCSE for tackling the rapidly rising levels of food insecurity, as the state was already directing vulnerable citizens to charitable food provision before COVID-19 (Garthwaite 2016a). As elsewhere in Europe, the crisis further cemented the controversial alliance between supermarkets and food charities, as many food support providers started or reinvigorated their collaboration with corporates (FEBA 2020; Power et al. 2020). In point of fact, during schools closure the UK government’s national distribution of supermarket vouchers worth £15 per week per eligible child suffered delays (Weale and Murray 2020) whereas the £20-a-week temporary increase to universal credit, though welcomed by charities, was not sufficient to prevent people on low incomes falling into hardship (Butler 2020a).^3^ Concurrently, the establishment of a £3.25 million fund for food redistribution organizations—de facto designed to strengthen the food supply of charities in a moment of mounting requests—seemed to confirm that responsibility was also—and perhaps mainly—on the shoulders of the third sector.

The case of Greater Manchester well exemplifies this process. Aware of the high poverty levels throughout the county (IGAU 2017), at the start of the epidemic the metropolis combined supply (of food aid coordinated by the local authority) with the organization of demand by setting up an application portal that gathered data on local food support providers and residents in need of support.^4^ Even more indicative was the opening of a council-led temporary food bank in Openshaw in partnership with both voluntary and private organizations. The service started door-to-door delivery of emergency parcels during the first weeks of the lockdown, totalling around 30,000 requests from March to May (Manchester City Council 2020). The unprecedented and generous effort put in place by public and private institutions has been justly celebrated by the press, which underlined the importance and the effectiveness of the third sector acting locally throughout the lockdown. This accolade is mostly based on commentary and anecdotes, however, and so might depict the evolution of the emergency response without attending to its problematic facets.

## Data and methods

### Data collection

This study is based on a combination of quantitative and qualitative analysis of data gathered by the author between June and August 2020.^5^ Survey data were collected to obtain standardized information on the characteristics of different food support providers and on the impact of COVID-19 on their operations. Starting from the open-data map of food support providers created by Greater Manchester Poverty Action (GMPA),^6^ I first extracted the datasheet containing contact details for 222 services connected with food aid throughout Greater Manchester. To this dataset I added the details of 26 food support providers found on the Mutual Aid Groups Map,^7^ and 9 others were reached by sending a link to the questionnaire in the GMPA newsletter,^8^ to get as close as possible to the statistical population. The contact database was then shared with a research agency that administered the questionnaire via CATI (Computer-Assisted Telephone Interviewing) or CAWI (Computer-Assisted Web Interviewing) once operators had spoken to directors or spokespersons.^9^ All calls were preceded by an email supplying a participant information sheet of the project, and information on how to give consent. At the end of the questionnaire, participants were invited to a one-hour Zoom interview with the author, to enable me to dig deeper into some aspects of the COVID-19 crisis and discuss the history and characteristics of the organization before the epidemic. Additional stakeholders working in the field of food aid (e.g. directors of charities that distribute funding, experts in food surplus redistribution, members of advocacy groups) were also recruited so I could gather additional evidence and discuss some of the patterns that emerged in previous conversations. All the interviews were recorded, anonymized, then transcribed verbatim. Participants who decided to fill in the questionnaire and/or participate in interviews received a small incentive in the form of charity donation.

### Sample selection and data analysis

The list of 257 food support providers contained 33 duplicates. Enquiries revealed that 41 did not provide food support or any type of aid; and 73 did not respond to several attempts to reach them, or their contact details were out of date. This latter group may have consisted of organizations that had to shut down for lack of volunteers or suitable spaces to reorganize support. Eventually, 55 directors/spokespersons participated in the survey and 55 either refused, or I could not secure a CATI/CAWI interview (50% of the ‘active’ population). Among the survey participants, 30 agreed to the follow-up interview via Zoom. The 12 additional stakeholders were additionally recruited by the author using personal contacts and snowball sampling.

**Fig. 1:**
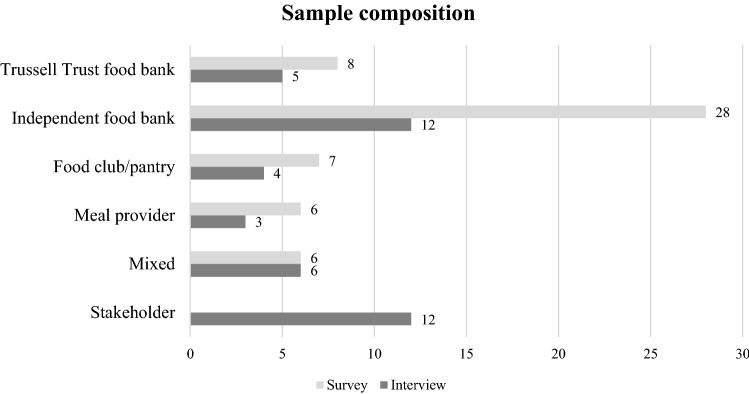
Composition of the sample for the survey and the interviews

Figure 1 illustrates the composition of the participants in the survey and the interviews. In both cases, this is rather heterogeneous, as they include both Trussell Trust and independent food banks (whether or not part of the IFAN), and also food clubs/pantries and warm meal providers.^10^ The ‘mixed’ category describes organizations combining more than one model (e.g. providing warm breakfasts and a food pantry). This achieves a composite picture of the situation by considering perspectives from organizations working in the same field but with different resources and modes of intervention.

Collection of the data proceeded simultaneously and interactively with analysis.^11^ In what follows, I will present descriptive statistics and some emblematic excerpts from the interviews (all names are pseudonyms) to illustrate three main outcomes of the response to the crisis: obstacles overcome, complications and restrictions.

## Findings

### Overcoming obstacles

The sudden increase in requests for emergency food could have put a strain on the capacity of food support providers to successfully meet the surge in demand. Table 1 shows, however, that during the first and most problematic weeks of the crisis, very few organizations had to turn eligible people away for any of the reasons listed. More than 80% of the respondents declared that they have never turned eligible people away on account of a lack of food stocks or organizational resources (volunteers and staff). Interestingly, almost no one responded that people were turned away due to a lack of valid food vouchers (92.7%). Studies before COVID-19 illustrated that people in need had to face bureaucratic hurdles and racialised calculations of deservingness in order to become eligible (de Souza 2019; May et al. 2019). During the crisis, however, it is possible that most providers renounced or reduced eligibility checks and procedures to streamline the distribution. Crucially, when forced to turn people away, users were often redirected to other providers with greater capacity, so that at no point during the crisis has there been an overall lack of food.

**Table 1:** How often organizations turned people away

During the last few weeks, how often have you needed to turn eligible people away due to lack of…	Food		Staff members		Volunteers		Lack of valid food voucher	
	n	%	n	%	n	%	n	%
Frequently	3	5.5	2	3.6	3	5.5	1	1.8
Occasionally	1	1.8	4	7.3	3	5.5	2	3.6
Rarely	3	5.5	3	5.5	4	7.3	1	1.8
Never	48	87.3	46	83.6	45	81.8	51	92.7
Total	55	100	55	100	55	100	55	100

Two concomitant factors contributed to this outcome. On one hand, repeated calls on the generosity of individuals and businesses (especially food retailers), as well as the UK government fund for food redistribution, injected the resources necessary to keep the food support system running. In fact, many organizations experienced an increase in donations of food (76.4% of sample) or money (67.3%) (see Table 2), with 85.5% (47 cases) reporting one of the two and more than half reporting both (32 cases, 58.2%). Interestingly, only four participants reported a fall in the nutritional value of the food distributed, whilst the majority (65.5%) reported no change. Surprisingly, 15 organizations saw an increase: while urgency could have resulted in a lower concern for food quality, it is likely that the rise in food and monetary donations had positive side effects on the heterogeneity of the food supplied to some providers and, in turn, on the overall quality of the groceries distributed.

**Table 2:** Changes in donations and nutritional value

Thinking about the following aspects of your organization, how have each of them changed since the beginning of the COVID-19 outbreak?	Volume of monetary donations		Volume of food donations		Nutritional value of the food	
	n	%	n	%	n	%
Decreased	8	14.5	10	18.2	4	7.3
Stayed the same	10	18.2	3	5.5	36	65.5
Increased	37	67.3	42	76.4	15	27.3
Total	55	100	55	100	55	100

On the other hand, providers demonstrated great resilience, with 89.2% of participants believing that the organization could recover and adapt quickly (Table 3). In fact, as soon as the lockdown was introduced by the government on 23 March 2020, organizations found ways to maintain existing food support channels. In some cases, as the following quotations from a food bank and a warm meal provider show, the transition was relatively smooth:

The first change was that we stopped doing hot drinks and we were still making the parcels as needed, but we were just minimising all the extra stuff that went around it. Then we changed to… people would just come to the front door, they wouldn’t come in to the building. And all of the parcels would be pre-made. And we would just distribute those, just take the voucher, give them a bag. So it was really… the contact was really minimal. And that was through maybe the end of March and the beginning of April. And then I think the first day that we decided... or the first day that we started doing deliveries, I think it was the 13th of April. Or it was, like, that week. So we started doing three days of deliveries per week… I think, to be honest, so far things have gone relatively smoothly. Which feels a little bit suspicious, it feels a little bit... we kind of haven’t really... I think we’re at a massive advantage because we have three of us [*Young people. Ed.*]*,* which means we've been able to respond to everything a lot more quickly. *Annie, Trussell Trust food bank*

So, once we became aware it was an issue, we sort of planned very quickly that we wouldn’t be able to carry on doing our normal sit-down meals with people. That’s before the lockdown came in or anything like that, we just knew that wasn’t going to happen so we did plan to be, to cook at the venue still and then for people to come and collect takeaways. We did that for one week and then we realised that that wasn’t really going to work very well and then the following week, after they introduced the lockdown bit anyway, so we wouldn’t have been able to carry on doing that anyway… We use the upstairs space that’s empty to sort it and bag it and we have delivery drivers going out to deliver it around to the people who have been referred to us. *Bill, Warm meal provider*

**Table 3:** Resilience to the COVID-19 crisis

How resilient do you feel your organization is likely to be against the COVID-19 crisis?	n	%
Very resilient	23	41.8
Fairly resilient	26	47.3
Not very resilient	3	5.5
Not at all resilient	1	1.8
Don't know/Refused	2	4.6
Total	55	100

Most food support providers were very agile in responding to the new necessities. As Annie’s words illustrate, the possibility of working with young colleagues who were not at high risk of COVID-19 complications, allowed one food bank to quickly modulate the service to respond to needs, to an extent that felt ‘a bit suspicious’, given the times. Similarly, many other food banks and smaller providers immediately set up points for picking up food and often temporary, door-to-door delivery services, while most food pantries remained open by making users observe the ‘same physical distancing’ and hygiene measures required in supermarkets. Some warm meal providers started to deliver cooked food or temporarily began distributing food parcels, using premises to accommodate food stocks, as Bill indicates. This transition was not always easy, for instance when providers were mainly people in their 60 s and 70 s. Even in such cases, however, some were able to find informal and ‘personal’ ways to remain open:

The two big problems that I’ve got with COVID is, first of all, my volunteers are all over 70. So they can’t continue. You know, they’ve got to... I’m healthy, I’m healthy as anything, as is my wife. But the simple statistic that says, if somebody over 70 gets it they’re 200 times more likely to die than if somebody at 50 gets it… So I’m guiding myself and my wife, and wonderful that my children are servicing us, and we’re getting the Tesco deliveries. That’s how we’re dealing with it personally… Now it’s just an odd... I’ve done a food bank thing with him. Somebody was critical for some food, I just sent my son to the shop to buy some food and then he got... well, he delivered that. *Chris, Independent food bank*

Like many other food support providers run by elderly volunteers, Chris’s food bank would have interrupted the service if not for his son, who took care of the delivery service and kept providing food for those in need. This illustrates the social commitment and dedication brought even to smaller and more informal food support providers, who probably explored all the alternatives to ceasing activity.

### Complications

The extraordinary success of food aid services in tackling the crisis did not come without its challenges. Although, as we have seen, many providers just switched mode of provision overnight to guarantee an adequate and timely supply of food, the pressure overload on the whole system created three main complications. First, many participants reported experiencing some shortage of staff members, and especially in volunteers. Only 52.7% declared that lack of staff had not been an issue and 40.0% that lack of volunteers was not an issue (Table 4). This is not surprising, as before the pandemic most independent food banks relied on five or more volunteers (Loopstra et al. 2019c). Although very few reported that shortage was a major issue, this may have implied a higher workload on the personnel at work during the crisis. As Delia, from an independent food bank, states:

The amount of food that we get donated has increased. People are aware of us now. We get a lot of publicity on Facebook, a lot of fundraising. But you know, the downside is that we’re getting a lot more people that need our service. We’re working with a lot of different agencies now. People come and collect emergency parcels for people and things like that. And we’ve had to change how we work, because we only ever used to attend the food bank on a Monday and a Friday, whereas because now a lot of our volunteers have had to self-isolate, we’ve had to spread the work out that we do over the whole week. Because there’s so much to do now with so few volunteers. *Delia, Independent food bank*

**Table 4:** Reported shortages

Is the organization short of:	Staff members		Volunteers		Food	
	n	%	n	%	n	%
1 Not at all	29	52.7	22	40.0	18	32.7
2	14	25.5	13	23.6	12	21.8
3	2	3.6	12	21.8	16	29.1
4	5	9.1	6	10.9	5	9.1
5 Very much so	5	9.1	2	3.6	4	7.3
Total	55	100	55	100	55	100

**Fig. 2:**
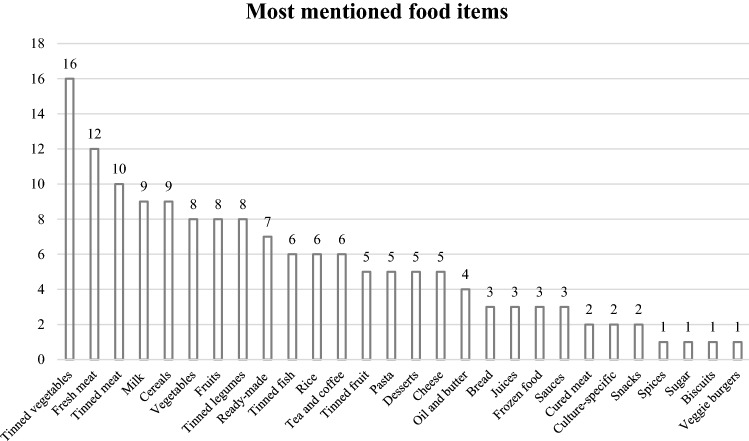
Food items most often lacked by organizations

Second, the fact that there was enough food to feed everyone does not mean that providers did not experience food shortages. As Table 4 indicates, only 32.7% stated that the organization experienced no problem of any sort, while more than 16% reported high levels of shortages. Figure 2 summarizes the items lacking from providers’ shelves: unsurprisingly, canned items are among the ones mentioned most often, probably because of the high demand for them, given their shelf-life. Interestingly however, lack of fresh products such as meat, cheese, fruit and vegetables were also reported, probably owing to short supply and/or costs and challenges in transportation (e.g. maintaining the cold chain).

Third, and connected, the higher pressure on the resources available to the providers has increased the uncertainty around their capacity to survive in the longer term—especially in view of a second wave. As Table 5 illustrates, 38.2% of food support providers reported that food stocks would last for four weeks or less (and 14.5% reported that cash reserves would last four weeks or less), while 5–8 weeks’ stock was reported by 7.3% and 5–8 weeks’ cash reserves by 9.1%.

**Table 5:** Number of weeks existing stock/reserves will last

How many weeks will your existing food stocks/cash reserves last at current levels of demand?	Food		Cash reserves	
	n	%	n	N
4 weeks or less	21	38.2	8	14.5
Between 5 and 8 weeks	4	7.3	5	9.1
More than 8 weeks	4	7.3	15	27.3
This is not an issue at present	19	34.5	16	29.1
Don't know	7	12.7	11	20.0
Total	55	100	55	100

Thus, although managing to address food poverty, providers have entered a precarious phase, especially the smallest organizations, often needing new venues to operate safely. The open-ended question in the survey ‘What are the immediate needs of the food provider?’ prompted these fears to be voiced:

Probably the funding to come in continuously. As far as the needs of food, we are purchasing as we go. There are other places like churches and community centres that are supporting us with constant donations. At the moment it is continuous support. That means donations and financial donations. Both are important right now, but it is very difficult to get things in bulk as there are shortages of things. *Eric, Food pantry*

Finance; the government money hasn’t come anywhere near us and even if we have written a bid for it the people reading it don’t understand charity finance. Also, the funding from central government was odd, large amounts were given to national organizations which don’t have the means to distribute locally and then other funders won’t fund the same areas even if you haven’t been successful. Which has meant very little money has come down for basic stuff like buying food. *Fiona, Independent food bank*

Immediate needs would be finance, I suppose. For staff and resources to reach our services, like PPE *[Personal protective equipment. Ed.]* and perspex stuff. We need funding for the charity to pay our staff and get them off furlough. *Geraldine, Independent food bank*

What we need is a permanent base of operations. We are using someone else’s premises that they have closed due to COVID and they have given to us. When they reopen we will have to move out. It is all space, we have just reviewed what we are doing, we are rethinking our overall strategy. *Harry, Warm meal provider*

This corresponds with several independent reports that came out during the same period, highlighting the risk of bankruptcy and that many UK charities expect income to reduce (Butler, 2020b) despite the £750 million package of support offered by the government in April (UK Government 2020b).

### Restrictions

Participants described both obstacles overcome in, and complications stemming from, the impact the virus outbreak had on several aspects of their organizations. As Table 6 sums up, a small minority of the respondents declared that COVID-19 had no impact at all on the financial stability (12.7%), the management (14.5%) or the functioning (9.1%) of their organization, while a substantial proportion (25.5%, 38.2%, 54.5% respectively) chose the opposite response category. As we have seen, the necessity to rapidly adapt to the new conditions to respond effectively to increasing demand came at the cost of uncertainty, precarity and higher workload. Yet more critical was the cumulative effect of such a frantic rearrangement on the ‘social ingredient’ at the core of any form of support. As reported elsewhere (May et al. 2019, p. 714), food charities are ambivalent spaces, ‘characterized by complex interconnectivities between shame and gratitude, stigma and acceptance, moral judgement and emotional support.’ Critical aspects related to the ways in which food support providers evaluate eligibility and distribute foods are therefore weaved with methods of offering emotional and practical support that are often crucial for many people in need. They can offer much more than food to those in hardship: ears to listen, arms that are willing to hug, voices to offer sympathy (and recommendations and financial advice, if asked for) and people who are inclined to befriend without judging or requiring something in return. The crisis, however, meant much of this could not be offered, and some was entirely sacrificed for the entire duration of the lockdown—quite likely, when it was needed the most. In fact, 56.4% of participants said that COVID-19 had a major effect on the social atmosphere of the provider, and only three organizations (5.5%) reported no change in social atmosphere at all.

**Table 6:** Impact of COVID-19 on various aspects of the organization

To what extent would you say COVID-19 has affected the following aspects of the organization	Financial stability		Management		Functioning		Social atmosphere	
	n	%	n	%	n	%	n	%
1 Not at all	7	12.7	8	14.5	5	9.1	3	5.5
2	8	14.5	6	10.9	4	7.3	4	7.3
3	16	29.1	8	14.5	3	5.5	10	18.2
4	10	18.2	12	21.8	13	23.6	7	12.7
5 Very much so	14	25.5	21	38.2	30	54.5	31	56.4
Total	55	100	55	100	55	100	55	100

This unfortunate transformation is poignantly described by Innes, who works in a community centre that offered cooked meals and that had to provisionally transform into a takeaway service:

Whatever we had we would give away to people as they said they needed it. And then COVID happened and our doors closed. When the lockdown came about, when it was first mooted, we had to close the doors. And in that time when, before the lockdown was announced, I think was a short period of time, we decided to provide breakfast and lunch as a takeaway service… So we were dealing with isolated people across the whole range, the isolated people would come to us pre-COVID because we were a safe space, we were a welcoming space, and because they weren't allowed to go in many cases to any other places… Our clients are very touchy-feely, you know. How the hell we could stop a lot of our clients wanting to give us a cuddle or pat us on the back, or shake our hand or whatever? You know, culturally, there were four guys who came from Sudan and anything we did, they wanted to shake our hands. Anything... we gave them a coffee, they wanted to shake my hand. And you know, the difficulty in not doing that now, the difficulty in not patting somebody on the back, the difficulty when you're seeing somebody crying, not to give them a hug, you know, it's... sorry, I'm going on a bit… COVID has sucked the life out of our centre. It’s taken what we were… *Innes, Mixed food provider*

This touching excerpt vividly conveys what was lost during the lockdown and how much the (indispensable) measures taken to contain the contagion conditioned the operations of the community centre. Innes makes it clear that food support providers have a social function, ‘sucked away’ by COVID-19, that goes well beyond the supply of meals or parcels. To an extent, even after the end of the first lockdown, many precautionary measures still mandated the avoidance of physical contact as freely as before COVID-19, thus leaving ‘the difficulty when you’re seeing somebody crying, not to give them a hug’ intact.

This dread was evoked by other interviewees. While the UK government was relaxing the first lockdown measures, Jane discussed how her food bank could reopen safely, and akin to Innes mused on the probable loss of the social aspect:

But then it’s a question of what PPE we use for the volunteers, and also whether we will be able to have sufficient volunteers if our older and less healthy people say ‘I can’t take that risk’. So it will take a lot of working out; so we may lose the social aspect, we may have to say ‘sorry, we can’t have people coming in and relaxing and sitting down’. All we could do is provide a bag at the door, either a takeaway meal on one day and takeaway tins on the other, or something like that. Which for us will be a great shame, because like I say, for a lot of these people, it’s not just the food. It’s the social aspect, and it’s somewhere to sit. And for people who are actually literally homeless or for people who are having to live with friends and relatives where it’s a bit cramped and a bit on top of each other and a bit frictional, that space to just sit and relax and chat won’t be there. *Jane, Independent food bank*

In a moment of widespread generosity and gigantic effort to feed the most disadvantaged, the hidden costs weighed primarily on the most symbolic—and yet very tangible—aspects of providing food (support). Although some organizations found other ways to offer that social function, for instance through messaging platforms and group phone calls, these were rather ersatz means of community-building rather than the real thing.^12^ In a sense, as ‘nutrition’ and ‘sustenance’ took precedence over ‘eating’ and ‘commensality’, physical distancing became, for many, social distancing.

## Concluding remarks

The spread of COVID-19 in the UK laid bare how much its retrenching welfare system relies on the third sector to support people in need. The case of food support provision is probably emblematic, as national and local government both leaned immediately on the existing third sector organizations, aware they could count on their embeddedness in particular areas. Making use of first-hand data gathered in Greater Manchester immediately after the peak of the first ‘wave’ of COVID-19, this paper aims to provide a detailed picture of how food support providers overcame obstacles, and the complications and restrictions that characterised their responses to the emergency. I found three main considerations.

First, most food support providers did not turn eligible people away, even when volunteer and staff capacity was lacking or because food in stock was in short supply. The capacity to improvise and quickly readapt to the new circumstances, coupled with the great generosity shown by individuals and companies and the efficient distribution of any food surplus, allowed them to respond promptly to the increasing demands for their services.^13^ For instance, many shifted logistics operations from food pick-up to food delivery to help users that were ‘shielding’ themselves. Although a number of food support providers were forced to shut down after the lockdown owing to a lack of volunteers and/or funds, the ‘parallel welfare’ put in place was able to absorb the most imperative needs that emerged after the outbreak. At the same time, such a successful endeavour points to the fact that despite the increased need, accessing food for distribution has not been a significant challenge. Echoing Lohnes’ (2020) work on the perverse governance mechanisms tying together food waste production and charitable food distribution in West Virginia, this finding illustrates that food support providers can unwittingly become woven into the fabric of a schizoid (food) economy based on scarcity logics, excess production and regulation of abundance; this is even more applicable in extraordinary times.

Second, complications that many organizations had to face included an increasing workload that fell on the shoulders of volunteers and staff members who continued to work while others were furloughed or shielding and also an increasing pressure to obtain sufficient food and financial resources to keep going. Interview participants, in particular, questioned whether food and monetary donations would steadily flow should additional lockdowns occur and often worried that the end of the furlough scheme and the possibility of a no-deal Brexit—both still plausible at the time of the interviews—would exacerbate the situation for many people who already struggled to make ends meet, especially over winter. This, in turn, could affect the capacity of many organizations to respond, especially the most fragile ones, some of which had less than two months’ food or cash resources at the time of the survey. With the benefit of hindsight, we now know that some of those concerns were partly alleviated with the Brexit withdrawal agreement and with the extension of the furlough scheme until April 2021. The recently announced repeal of the £20-per-week uplift in universal credit from October 2021, however, casts a shadow on the future prospects of low-income families, who will see a drop in their income precisely when the country will hit its peak unemployment rate (The Guardian 2021).

Third, and perhaps most importantly, the necessity of maintaining the supply of food at all costs came with important restrictions. The lockdown measures that followed the first ‘wave’ not only affected the financial stability and the management of these organizations, but actually undermined their ability to provide crucial ancillary services. Unfortunately, physical distancing measures and shifts in logistics forced operators to change, and sometimes to suspend, engagement and interaction with people asking for support, which often meant weakening and devitalizing the very nature of that relationship. One might wonder whether, if the welfare state had been better able to take care of the most disadvantaged members of our societies, the many VCSE organizations solely dedicated to distributing food support during the crisis could have redirected their efforts towards many other forms of social inclusion, tailored on other needs of their users.

As recently pointed out by Dickinson (2020, p. 589), “it is not our supply systems that are breaking down and causing hunger, but our systems for ensuring people can access the food that exists which have been broken for a long time.” COVID-19 tested the strengths and weaknesses of our food provision system—a test we may need to get used to considering that short disruptive shocks characterise the Anthropocene (Benton 2020)—and showed what needs to be fixed to build a more just, inclusive, and sustainable future (Anderson 2020). The central role played by food charities and advocacy groups during the crisis could represent an important opportunity to re-orient policies to nourish communities and protect agricultural livelihoods and labour, as there is now more awareness on how food is produced, distributed, and eventually arrives—or not—on people’s plates (Graddy-Lovelace 2020).

To conclude, some limitations should be addressed, and this study suggests future directions for research. One of the central objectives of this paper is to set down the extreme complexity of the emergency response deployed during the COVID-19 crisis. In my view, this could help us get an overarching understanding of the successes, the obstacles faced by and the prospects of food support provision in Greater Manchester and possibly beyond, as well as grasping how crucial its role has been (and continues to be) in such a harsh period. At the same time, I am fully aware that some organizations may have experienced only bits and pieces of what is written here, and that their unique experiences could be partially mischaracterized. Looking in detail, it is plausible that food banks, warm meal providers and food pantries had to overcome different challenges while reorganizing to face the crisis. However, trying to identify common patterns allows us to zoom in on idiosyncrasies within the sector at a later stage. Future case studies focusing on single organizations could take the findings here presented as a benchmark and dig deeper into how specific actions were taken. Furthermore, for lack of data I could not focus on the role played by Mutual Aid Groups (MAGs), which were often working side by side with many food aid services and providing complementary forms of support thanks to their flexibility and informality (Reicher and Stott 2020). Systematic research on this elusive yet crucial part of the emergency response is still lacking and would further contribute to a thorough understanding.

On the methodological side, I should stress that, despite the satisfactory response rate and good variation in both the type and the location of food support providers who took part in both the survey and the interviews, positive selection bias could have occurred, as organizations experiencing operational difficulties may have avoided taking part. If this was the case, it is possible that the research overestimates providers’ success in overcoming obstacles and underestimates the challenges met by these organizations in taking action. Moreover, it is likely that the UK experienced regional variations in the organization of the food emergency response that cannot be observed by looking solely at one metropolitan county, however varied it may be. This points to the lack of survey data available on food support provision at the national (and supranational) level. If a major survey of food support providers were available, the monitoring during the first months of COVID-19 would have been much faster, more valid, and more reliable. To date, survey data have usually been collected by food charities or food surplus distributors for internal monitoring purposes, only shared with some researchers (e.g. Loopstra et al. 2019b) and understandably subject to non-disclosure agreements Sometimes—as for this article—researchers collect their own survey data, hence facing high marginal costs that could be greatly reduced if economies of scale could be exploited; this approach also increases survey fatigue for respondents who are repeatedly asked to fill in similar questionnaires. The paramount role now played by food support providers in the UK, and more generally in Europe, requires a more integrated approach to data collection that transcends single types of providers or networks—and perhaps even countries—and that allows researchers access on demand to harmonised data on several aspects of food charities (and their users) and their evolution over time. This would allow researchers to analyse in greater detail how different organizations obtain and share resources and to highlight inequalities in the access to donation networks, depending on location and the type of food support provision offered (May et al. 2020). At the same time, food charities could count on the skills and knowledge of a much wider pool of experts, as well as on a detailed data resource, to help them advocate the right to obtain good food in a just food system.

## Supplementary Information

Below is the link to the electronic supplementary material.

Supplementary file1 (DOCX 15 kb)

## Abbreviations

VCSE: The voluntary, community and social enterprise sector
FEBA: European food banks federation
IFAN: Independent food aid network
SDG: Sustainable development goals
GMPA: Greater manchester poverty action
MAG: Mutual aid group
CATI: Computer assisted telephone interviewing
CAWI: Computer assisted web interviewing

## Footnotes

1 Interestingly, the same phrase was used by the Conservative–Liberal Democrat coalition government to justify the austerity measures put in place during the European sovereign debt crisis in 2010 (see Flanders 2010).

2 More than a win–win situation, the interdependence between food surplus and food poverty should be seen as a zero-sum game: that is, the utility each participant gains or loses is exactly balanced by the utility the other participants lose or gain. While donating food surplus certainly represents a gain for companies—they avoid paying for disposal and improve their brand reputation—for most poor individuals receiving surplus food entails a loss of personal dignity, self-esteem and freedom of choice.

3 This is further suggested by charities’ plea to continue the £20-a-week COVID boost, as according to a recent Trussell Trust report its withdrawal would pull 700,000 more people into poverty (Weekes et al. 2020).

4 See https://surveys.tfgm.com/snapwebhost/s.asp?k=158748391626. Accessed 29 Mar 2021.

5 The research obtained ethical clearance from the University of Manchester Ethics Committee. The ethics application with details on data processing and security is available upon request.

6 The map is available at the following link: https://www.gmpovertyaction.org/maps/ (Accessed 29 Mar 2021). Although data are kindly provided at no charge by the GMPA, I informed the charity about the study and its members, along with other advocacy groups and charities, contributed to the questionnaire layout before starting data collection. The author’s preliminary reports on the data are also available on the GMPA’s website.

7 See https://covidmutualaid.org/local-groups/. Accessed 29 Mar 2021.

8 This strategy allowed to reach also those food support providers that asked not to be included in the GMPA map but regularly receive the newsletter.

9 The full questionnaire is available upon request. The dataset is embargoed for two years, and will be made freely available from October 2023. Preliminary access to aggregate data is available upon request.

10 Food clubs/pantries provide access to groceries—usually every week—for payment of a small subscription fee, while warm meal providers are organizations that distribute cooked meals or operate free access canteens.

11 Table A1 in the supplemental material summarises the questions and response categories used in the data analysis.

12 Social networks and messaging platforms have helped many people cope with the lockdown, across the socioeconomic spectrum. At the same time, COVID-19 has shed light on the magnitude of digital inequalities in the UK (Watts 2020), which in turn implies that switching social support online would not have the same reach as offline support.

13 Since food bank usage is a poor indicator of food insecurity (Loopstra and Tarasuk 2015), this is no guarantee that people did not experience hunger or did not cut back on other essentials in order to eat. It is possible that perspectives gained from ethnographic engagement or interviews with people who tried to access the service could unveil difficulties in obtaining food aid during the crisis.
