# Supplementary material for: Food support provision in COVID-19 times: a mixed method study based in Greater Manchester
Source: Agric Human Values. 2021 Apr 26;38(4):1201–13. doi: 10.1007/s10460-021-10212-2 (PMC8072744; doi:10.1007/s10460-021-10212-2)
Supplement: Supplementary file 1 — Supplementary file1 (DOCX 15 kb) [file 10460_2021_10212_MOESM1_ESM.docx]

**Supplementary material**

| **Original question** | **Response categories** |
| --- | --- |
| What type of food support provider are you? | Trussell Trust food bank,  Independent food bank,  Food club,  Food pantry,  Meal provider,  Other (specify),  We are not a food support provider |
| On a scale from 1 to 4, where 1 is “Frequently” 2 is “Occasionally”, 3 is “Rarely” and 4 is “Never”, during the last few weeks, how often have you needed to turn eligible people away…   1. Due to lack of food? 2. Due to lack of staff capacity? 3. Due to lack of volunteer capacity? 4. Due to a lack of valid food vouchers? | 1 = Frequently,  2 = Occasionally,  3 = Rarely,  4 = Never |
| Thinking about the following aspects of your organisation, how have each of them changed since the beginning of the COVID-19 outbreak?   1. Volume of monetary donations 2. Volume of food donations 3. Nutritional value of the food | Decreased,  Stayed the same,  Increased |
| Overall, how resilient do you feel your organisation is likely to be against the COVID-19 crisis? | Not at all resilient,  Not very resilient,  Fairly resilient,  Very resilient,  Don’t know,  Refused |
| Could you briefly tell me in your own words what have been the major changes in the overall organization after the COVID-19 outbreak? | Open response |
| On a scale from 1 to 5, where 1 is “Not at all” and 5 is “Very much so”, how much is the organization short on   1. Staff members? 2. Volunteers? 3. Food? | 1 = Not at all to  5 = Very much so |
| If the organization is short on food, what food items do you need the most? | Open response (recoded) |
| Roughly, how many weeks will your existing food stocks last at current levels of demand? | Open response (numeric) |
| Roughly, how many weeks will your existing cash reserves last at current levels of demand? | Open response (numeric) |
| To what extent would you say COVID-19 has affected the following…   1. Financial stability of the food support provider 2. Management of the food support provider 3. Functioning of the food support provider 4. Social atmosphere of the food support provider | 1 = Not at all to 5 = Very much so |
| Could you briefly tell me, in your own words, what are the immediate needs of your food support provider? | Open response (recoded) |

**Table A1. Original questionnaire and response categories used in the analysis.**
